# Smartphone sensors for evaluating COVID-19 fear in patients with cancer: a prospective study

**DOI:** 10.3389/fpubh.2023.1308003

**Published:** 2024-01-05

**Authors:** Gabrielė Kasputytė, Gabrielė Jenciūtė, Nerijus Šakinis, Inesa Bunevičienė, Erika Korobeinikova, Domas Vaitiekus, Arturas Inčiūra, Laimonas Jaruševičius, Romas Bunevičius, Ričardas Krikštolaitis, Tomas Krilavičius, Elona Juozaitytė, Adomas Bunevičius

**Affiliations:** ^1^Faculty of Informatics, Vytautas Magnus University, Kaunas, Lithuania; ^2^Faculty of Political Science and Diplomacy, Vytautas Magnus University, Kaunas, Lithuania; ^3^Oncology Institute, Lithuanian University of Health Sciences, Kaunas, Lithuania; ^4^ProIT, Vilnius, Lithuania; ^5^Department of Neurology, Columbia University Vagelos College of Physicians and Surgeons, New York, NY, United States

**Keywords:** cancer, COVID-19, digital phenotyping, patients’ behavior, fear, sensors

## Abstract

**Objective:**

This study aimed to analyze the association between the behavior of cancer patients, measured using passively and continuously generated data streams from smartphone sensors (as in digital phenotyping), and perceived fear of COVID-19 and COVID-19 vaccination status.

**Methods:**

A total of 202 patients with different cancer types and undergoing various treatments completed the COVID-19 Fears Questionnaire for Chronic Medical Conditions, and their vaccination status was evaluated. Patients’ behaviors were monitored using a smartphone application that passively and continuously captures high-resolution data from personal smartphone sensors. In all, 107 patients were monitored for at least 2 weeks. The study was conducted between August 2022 and August 2023. Distributions of clinical and demographical parameters between fully vaccinated, partially vaccinated, and unvaccinated patients were compared using the Chi-squared test. The fear of COVID-19 among the groups was compared using the Mann–Whitney and the Kruskal–Wallis criteria. Trajectories of passively generated data were compared as a function of fear of COVID-19 and COVID-19 vaccination status using local polynomial regression.

**Results:**

In total, 202 patients were included in the study. Most patients were fully (71%) or partially (13%) vaccinated and 16% of the patients were unvaccinated for COVID-19. Fully vaccinated or unvaccinated patients reported greater fear of COVID-19 than partially vaccinated patients. Fear of COVID-19 was higher in patients being treated with biological therapy. Patients who reported a higher fear of COVID-19 spent more time at home, visited places at shorter distances from home, and visited fewer places of interest (POI). Fully or partially vaccinated patients visited more POI than unvaccinated patients. Local polynomial regression using passively generated smartphone sensor data showed that, although at the beginning of the study, all patients had a similar number of POI, after 1 week, partially vaccinated patients had an increased number of POI, which later remained, on average, around four POI per day. Meanwhile, fully vaccinated or unvaccinated patients had a similar trend of POI and it did not exceed three visits per day during the entire treatment period.

**Conclusion:**

The COVID-19 pandemic continues to have an impact on the behavior of cancer patients even after the termination of the global pandemic. A higher perceived fear of COVID-19 was associated with less movement, more time spent at home, less time spent outside of home, and a lower number of visited places. Unvaccinated patients visited fewer places and were moving less overall during a 14-week follow-up as compared to vaccinated patients.

## 1 Introduction

Cancer is the leading cause of morbidity and mortality worldwide (1–3). The prognosis of cancer patients is improving due to earlier detection and increasing availability of effective therapies, such as immunotherapy and targeted therapies, which has resulted in a steadily increasing number of cancer survivors (4–7). Precise monitoring of cancer patients and survivors is important for the detection and prevention of treatment complications and disease progression (8), and there is an urgent need to develop widely accessible, automated, and evidence-based cancer patient monitoring systems (9). The increasing penetration of smartphones, coupled with embedded sensors and modern communication technologies, makes smartphones an attractive technology for continuous and remote monitoring of an individual’s health and well-being with negligible additional costs (10). Digital phenotyping allows continuous, spatially, and temporally precise monitoring of an individual in his/her natural environment by using passively generated data from smartphone sensors (11, 12). This approach has been shown to identify treatment complications and disease trajectories of patients with cancer (13, 14).

The COVID-19 pandemic has disproportionally affected patients with cancer due to their often immunocompromised status and frequent hospital visits making them vulnerable to contracting COVID-19 infection and experiencing severe and complicated COVID-19 (15). The coronavirus case-fatality rate is higher in cancer patients when compared to the general population (16). Numerous strategies were used to mitigate COVID-19 risk in patients with cancer, including the transformation of oncology services, personal protective equipment and personal space modifications, and limited family attendance during chemotherapy sessions (17). Vaccination provides an important protection from COVID-19; however, COVID-19 vaccines are less effective in patients with cancer (10). Cancer patients experience significant fear of COVID-19, which can further lead to adverse mental health outcomes that can adversely impact their quality of life (18, 19). Therefore, the identification of behavioral impacts of the COVID-19 pandemic on patients with cancer could help to further improve the quality of life of these patients during the COVID-19 pandemic and beyond. While the global COVID-19 pandemic has ended, the COVID-19 virus continues to circulate and societies are still facing adverse mental health and behavioral impacts of COVID-19 such as post-pandemic and re-entry anxiety (20, 21).

Prior studies on the psychological impact of the COVID-19 pandemic on cancer patients typically used self-reported questionnaires. Digital phenotyping approaches are increasingly tested across mental health, including post-traumatic stress disorder (22), depression (23), and schizophrenia (14). Digital phenotyping allows for monitoring and predicting psychological states using passively generated data from smartphone sensors (23). Better characterization of the COVID-19 pandemic’s impact on the behavior of cancer patients in their natural environments and its possible association with psychological health status could be used to identify cancer patients who are vulnerable to experiencing negative psychological and behavioral impacts of the COVID-19 pandemic.

In this study, we analyzed the association between the behavior of cancer patients, measured using passively and continuously generated data streams from smartphone sensors (as in digital phenotyping), and perceived fear of COVID-19 and COVID-19 vaccination status. We sought to explore if the distributions of passively collected data resembling physical and social activity differ as a function of subjective COVID-19 fear and COVID-19 vaccination status after the COVID-19 pandemic.

## 2 Materials and methods

### 2.1 Study population

Consecutive patients undergoing treatment for cancer at the Hospital of Lithuanian University of Health Sciences Kaunas Clinics in Kaunas, Lithuania were invited to participate in this prospective observational study. The study inclusion criteria were diagnosis of any cancer, current active treatment for cancer or surveillance, ability to understand Lithuanian, ownership of a smartphone device that could support the smartphone application, the age of 18 years or older, and ability to provide informed consent. There were no exclusion criteria with regard to cancer diagnosis or type of active treatment at the time of invitation to participate in the study. The study period was from August 2022 to August 2023.

The study protocol and its consent procedure were approved by the Kaunas Regional Bioethics Committee (14th of April 2022, protocol number BE-2-31), Kaunas, Lithuania. All participants gave signed informed consent before inclusion in the study.

### 2.2 Study procedures

At the initial visit, the study participants were registered in the study portal^1^ and the LAIMA application was installed on their smartphones. Participants were provided with personal passwords and were informed about the procedures in case they decided to withdraw from the study. Moreover, participants completed the COVID-19 Fears Questionnaire for Chronic Medical Conditions, which was deployed by the LAIMA application. Monitoring and data collection of passively generated data via smartphone sensors and actively collected questionnaire data via the LAIMA application were conducted daily.

### 2.3 Demographic and clinical characteristics

Demographic information about the patient’s age, sex, education, marital status, and occupation as well as information about cancer diagnosis, location, and currently used medications were collected. Moreover, currently active cancer treatments, including biological therapy, chemotherapy, hormone treatment, immunotherapy, and radiotherapy, were recorded. We also gathered information about all previous cancer treatments (surgery, biological therapy, chemotherapy, hormone treatment, immunotherapy, and radiotherapy). The ECOG performance status was evaluated.

All clinical and demographic data were recorded at the LAIMA platform by board-certified oncologists.

### 2.4 COVID-19 vaccination status

Patients in this study were grouped by their COVID-19 vaccination status. There were three types of vaccination status: fully vaccinated, partially vaccinated, and unvaccinated. When at least 2 weeks had passed since receiving the second dose of the COVID-19 vaccine, a patient was considered fully vaccinated. Partially vaccinated status was mentioned when more than 2 weeks had passed since the first dose of the COVID-19 vaccine, which includes two doses. If the patient had not received any doses of the COVID-19 vaccine, they were considered unvaccinated.

### 2.5 COVID-19 fears questionnaire for chronic medical conditions

Fears related to the COVID-19 pandemic were evaluated using the COVID-19 Fears Questionnaire for Chronic Medical Conditions (COVID-19 fears) (24), which was previously translated and validated in Lithuania (25). The questionnaire was delivered via the LAIMA application. The questionnaire includes 10 statements that describe a patient’s experience on a typical day in the last week on a 5-point numerical scale ranging from 1 (not at all) to 5 (extremely). Scores were linearly transformed to a scale ranging from 0 to 100, with a higher score indicating greater COVID-19 fear. Patients were dichotomized into two groups based on the total score. Patients who scored ≥50 were considered as a group that was afraid of COVID-19 and those who scored <50 were considered as a group that did not experience fear of COVID-19.

### 2.6 Passive data analysis

Passively generated data from smartphone sensors (passive data) were collected using the LAIMA application, which was developed based on the open-source Beiwe platform.^2^ LAIMA application is supported by iOS and Android operating systems. Along with the smartphone application, we also developed a digital platform that was used to register and remotely monitor patients. The LAIMA application and platform are compliant with the European Union General Data Protection Regulation (GDPR) requirements (26). Cloud services were utilized for data storage. Only 107 of 202 patients were monitored for at least 2 weeks. To avoid deviations in sensor values due to different operating systems or smartphone models, the values were normalized. In general, no significant difference in data validity between different operating systems was observed, and it could be concluded that data transmission problems were not related to the operating system. There were several possible reasons for transmission problems: (a) the application was deleted (data were no longer being sent), (b) data transmission permissions were disabled (data were no longer being sent), (c) phone battery optimization was enabled (not all data were being sent), (d) the phone was not used (unchanging coordinates were being sent), and (e) the phone was turned off (data were not being sent). Since all patients participated in the study voluntarily, turning off the apps or not using the phone was unavoidable. However, when data transmission malfunctions were noticed, the researchers contacted the patients and provided all necessary information to resume data collection.

Accelerometer, GPS, log files, and Power State data were used, analyzed, and aggregated. Accelerometer data consisted of a timestamp, *x* coordinate, *y* coordinate, and *z* coordinate. All values of coordinates were aggregated by seconds and normalized by the following formula:


$$x_{\mathrm{norm}}=\frac{x}{\max|x|}\text{,}$$

where $x_{\mathrm{norm}}$ is the normalized value, x is the real value, and max|x| is the maximum of the absolute value.

Using accelerometer data, the duration of activity was determined.

GPS data consisted of a timestamp, longitudinal coordinate, latitudinal coordinate, and altitude. All values of GPS were aggregated by seconds. The distances between the observed coordinates were calculated using the *pointDistance* function in the *raster* library, which evaluates the geographical distance between two World Geodetic System ellipsoid points (27). GPS data were used to estimate the duration of movement, the time spent in the most frequently visited place (supposedly home), the average distance to that place, and the number of places of interest (POI) per day.

Power State data consisted of a timestamp and event (screen on/screen off). The differences between different event timestamps were calculated and aggregated by day.

Log files allowed us to determine how much data accumulated per day. The number of created files during the day was indicated by the records “Create new data files.” With this additional log information, the duration of the activity, movement, time spent at home, and screen time were normalized and the ratio was calculated, considering the duration of collected data and, thus, protecting against data flow problems.

The details of aggregated and newly created variables are described in Table 1.

**Table 1:** Variables of passive data.

Variable	Sensor used to create the variable	Description
Activity duration ratio	Accelerometer	The ratio of physical activity duration per day. The calculation followed these steps: (1) Accelerometer data variance was calculated for each minute: $v\left(d,m\right)=v_{x}\left(d,m\right)+v_{y}\left(d,m\right)+v_{z}\left(d,m\right)\text{,}$ where $v_{x}$ is the variance of *x* coordinate values, $v_{y}$ is the variance of *y* coordinate values, $v_{z}$ is the variance of *z* coordinate values, *d* denotes day, and *m* denotes minute. (2) Periods of unchanging phone status were identified: If the variance of coordinates was less than a threshold of 0.0001 g^2^, it was assumed that the phone state did not change for that minute (14). (3) All periods of steady phone states were summed up.
Movement duration ratio	GPS	The ratio of movement duration per day (walking/jogging). The movement was indicated as the changes in coordinates, where the calculated speed was less than 10 km/h.
Time spent at home ratio	GPS	The ratio of time spent at the most frequently visited place, supposedly home. The identification of home followed these steps: (1) the most frequent values of coordinates in the time range of 2 a.m. and 5 a.m. were found; (2) these coordinates were assigned to “home” coordinates; and (3) a 10-m radius was attributed to the same place.
Distance to home	GPS	The average distance to home per day (km). The distance to the coordinates, which were assigned to the home, was calculated using the *pointDistance* function (*raster* library, RStudio).
Places of interest (POI)	GPS	The number of places of interest (POI) per day. POI were described as a place in which a patient spends not less than 30 min. The location was evaluated from coordinates. A 100-m radius was attributed to the same place.
Screen time ratio	Power State	The ratio of smartphone screens on duration per day.

## 3 Results

### 3.1 Clinical and demographic data of the patients

A total of 202 patients (69% women, mean age 54.2 ± 17.4) with cancer were included in the study (Table 2). The majority of patients had academic degrees (62%), were married (72%), lived in urban areas (74%), were employed (58%), and had Android smartphones (85.1%). The most common cancer types were breast (18.9%), prostate (12.5%), cervix (11.9%), digestive tract (11.9%), and hematological (10.5%), followed by uterine (9.8%), lung (8.4%), mouth (4.2%), and other types (11.9%). Less than half of the patients had a history of cancer surgery (42.6%), biological therapy (8.5%), chemotherapy (34.2%), hormone treatment (13.4%), or immunotherapy (5.5%). Slightly more than half of the patients were undergoing chemotherapy (50.5%), 17.4% were undergoing hormone treatment, 13.4% were undergoing biological therapy, and only 4.9% of patients were undergoing immunotherapy. Half of the patients were undergoing radiotherapy, while the most common radiotherapy localization was pelvis (33.7%) and prostate (17.8%), followed by lungs (7.9%), breasts (5.9%), head–neck (5.9%), and other localizations (28.8%). Cisplatin was used to treat 16.8% of patients. The majority of patients were fully active and were able to carry on all pre-disease performances without restriction (87.6%).

**Table 2:** Patient characteristics by COVID-19 vaccination status.

Clinical and demographic parameter	Characteristic	Fully vaccinated, *n* = 143 (71%)	Partially vaccinated, *n* = 26 (13%)	Unvaccinated, *n* = 33 (16%)	$\chi^{2}$	*p*-value
Age	18–25	5 (3.5)	1 (3.9)	1 (3.0)	6.884	0.331
	26–40	13 (9.1)	5 (19.2)	7 (21.2)		
	41–64	78 (54.5)	15 (57.7)	18 (54.6)		
	≥65	47 (32.9)	5 (19.2)	7 (21.2)		
Sex	Male	57 (39.9)	15 (57.7)	12 (36.4)	3.322	0.189
	Female	86 (60.1)	11 (42.3)	21 (63.6)		
Education	With academic degree	89 (62.2)	15 (57.7)	19 (57.6)	0.372	0.829
	Without academic degree	54 (37.8)	11 (42.3)	14 (42.4)		
Marital status	Single	13 (9.1)	4 (15.4)	3 (9.1)	4.814	0.567
	Married	103 (72.0)	17 (65.4)	21 (63.6)		
	Divorced	15 (10.5)	2 (7.7)	7 (21.2)		
	Widowed	12 (8.4)	3 (11.5)	2 (6.1)		
Living area	Urban area	106 (74.1)	17 (65.4)	24 (72.7)	0.848	0.654
	Rural area	37 (25.9)	9 (34.6)	9 (27.3)		
Occupation and employment status	Employed	83 (58.0)	13 (50.0)	19 (57.6)	6.221	0.398
	Unemployed	9 (6.3)	3 (11.5)	5 (15.2)		
	Retired	40 (28.0)	6 (23.1)	8 (24.2)		
	Other	11 (7.7)	4 (15.4)	1 (3.0)		
Smartphone operating system	Android	120 (83.9)	24 (92.4)	28 (84.8)	9.263	0.055
	iOS	23 (16.1)	1 (3.8)	5 (15.2)		
	Other	0 (0)	1 (3.8)	0 (0)		
Cancer type	Breast	27 (18.9)	1 (3.8)	5 (15.2)	33.33	**0.015**
	Cervix	17 (11.9)	4 (15.4)	11 (33.3)		
	Prostate	18 (12.5)	3 (11.5)	5 (15.1)		
	Digestive tract	17 (11.9)	3 (11.5)	3 (9.1)		
	Lungs	12 (8.4)	3 (11.5)	2 (6.1)		
	Uterus	14 (9.8)	1 (3.8)	1 (3.0)		
	Hematological	15 (10.5)	1 (3.8)	1 (3.0)		
	Mouth	6 (4.2)	1 (3.8)	2 (6.1)		
	Other	17 (11.9)	9 (34.6)	3 (9.1)		
History of COVID-19	Yes	53 (37.1)	12 (46.2)	20 (60.6)	6.3	**0.042**
	No	90 (62.9)	14 (53.8)	13 (39.4)		
History of cancer surgery	Yes	65 (45.5)	11 (42.3)	10 (30.3)	2.518	0.283
	No	78 (54.5)	15 (57.7)	23 (69.7)		
History of biological therapy	Yes	13 (9.1)	3 (11.5)	1 (3.0)	1.655	0.437
	No	130 (90.9)	23 (88.5)	32 (97.0)		
History of chemotherapy	Yes	55 (38.5)	7 (26.9)	7 (21.2)	4.241	0.119
	No	88 (61.5)	19 (73.1)	26 (78.8)		
History of hormone treatment	Yes	19 (13.3)	3 (11.5)	5 (15.2)	0.166	0.92
	No	124 (86.7)	23 (88.5)	28 (84.8)		
History of immunotherapy	Yes	8 (5.6)	3 (11.5)	0 (0)	3.781	0.151
	No	135 (94.4)	23 (88.5)	33 (100.0)		
Current chemotherapy	Yes	74 (51.7)	11 (42.3)	17 (51.5)	0.8	0.67
	No	69 (48.3)	15 (57.7)	16 (48.5)		
Current hormone treatment	Yes	27 (18.9)	3 (11.5)	5 (15.2)	0.958	0.619
	No	116 (81.1)	23 (88.5)	28 (84.8)		
Current biological therapy	Yes	20 (14.0)	4 (15.4)	3 (9.1)	0.659	0.718
	No	123 (86.0)	22 (84.6)	30 (90.9)		
Current immunotherapy	Yes	8 (5.6)	3 (11.5)	1 (3.0)	1.988	0.369
	No	135 (94.4)	23 (88.5)	32 (97.0)		
Current radiotherapy	Yes	69 (48.3)	12 (46.2)	20 (60.6)	1.813	0.403
	No	74 (51.7)	14 (53.8)	13 (39.4)		
Radiotherapy localization	Pelvis	22 (31.9)	2 (16.7)	10 (50.0)	20.336	0.404
	Prostate	12 (27.5)	1 (8.3)	5 (25.0)		
	Lungs	6 (8.7)	2 (16.7)	0 (0.0)		
	Breasts	4 (5.7)	1 (8.3)	1 (5.0)		
	Head–neck	3 (4.3)	1 (8.3)	2 (10.0)		
	Other	22 (31.9)	5 (47.7)	2 (10.0)		
Currently on cisplatin	Yes	19 (13.3)	4 (15.4)	11 (33.3)	5.944	0.051
	No	124 (86.7)	22 (84.6)	22 (66.7)		
ECOG	0	123 (86.0)	23 (88.5)	31 (93.9)	1.788	0.774
	1	19 (13.3)	3 (11.5)	2 (6.1)		
	2	1 (0.7)	0 (0)	0 (0)		

Bold values mean statistical significant differences.

The majority (70.8%) of patients were fully vaccinated for COVID-19, while 13% of patients were partially vaccinated and 16% of patients were not vaccinated for COVID-19. History of COVID-19 was more common in patients who were not vaccinated for COVID-19 (60.6%) when compared to partially (46%) and fully (37%) vaccinated patients (*p* = 0.042). COVID-19 vaccination status was different as a function of cancer type (*p* = 0.015). One-third of patients with cervix cancer (33.3%) were unvaccinated, whereas 34.6% of patients with unspecified cancer types were partially vaccinated. Other demographic and clinical characteristics were similar as a function of the COVID-19 vaccination status.

### 3.2 Fear of COVID-19 and vaccination status

Fear of COVID-19 was statistically significantly different according to COVID-19 vaccination status (*p* = 0.024). The higher score of COVID-19 fear was noticed in patients who were fully vaccinated (23.51 ± 19.95) or unvaccinated (20.84 ± 14.82) rather than partially vaccinated patients (11.47 ± 10.60). Patients who were currently treated with biological therapy reported higher scores on the COVID-19 fear questionnaire (28.74 ± 18.11) than those who were not (20.87 ± 18.97) (*p* = 0.043). COVID-19 fear was not different as a function of other demographic and clinical characteristics.

### 3.3 Passively generated data and COVID-19 fear

**Figure 1:**
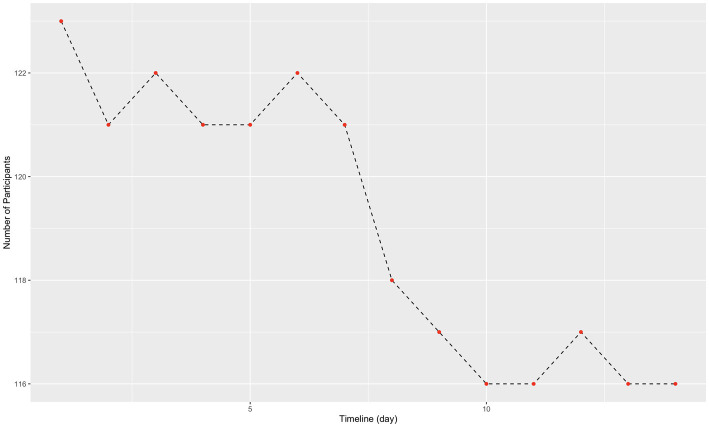
Follow up data collection in the trial.

Patients who had at least 2 weeks of observations using their smartphone sensors were included. These data were aggregated, and a mean of 2 weeks was used in the analysis. Overall, 116 patients had at least 2 weeks of passively generated data (Figure 1). Among them, 31 patients reported fear and 85 patients did not report fear of COVID-19 (Table 3). The duration of movement was statistically significantly higher between patients who feared COVID-19 and those who did not fear COVID-19 (*p* = 0.008). Furthermore, patients who feared COVID-19 spent more time at home (*p* = 0.047), visited places at shorter distances from home (*p* = 0.038), and visited fewer places of interest (*p* = 0.047) than patients who did not fear COVID-19.

**Table 3:** Statistical significance difference in activity characteristics based on fear of COVID-19.

Variables of physical and social activity	Mean ± SD	Statistical value
Movement duration ratio (range [0;1])		
Fear of COVID-19 score ≥ 50	0.105 ± 0.053	**P = 0.008**
Fear of COVID-19 score < 50	0.071 ± 0.047	U = 1,481
Distance to home (km)		
Fear of COVID-19 score ≥ 50	8.3 ± 19.102	**P = 0.038**
Fear of COVID-19 score < 50	15.7 ± 24.201	U = 813
Time spent at home ratio (range [0;1])		
Fear of COVID-19 score ≥ 50	0.744 ± 0.226	**P = 0.047**
Fear of COVID-19 score < 50	0.633 ± 0.254	U = 1,386
Number of POI		
Fear of COVID-19 score ≥ 50	2.711 ± 1.222	**P = 0.047**
Fear of COVID-19 score < 50	3.461 ± 1.613	U = 826
Activity duration ratio (range [0;1])		
Fear of COVID-19 score ≥ 50	0.552 ± 0.146	P = 0.993
Fear of COVID-19 score < 50	0.538 ± 0.182	U = 381
Screen time ratio (range [0;1])		
Fear of COVID-19 score ≥ 50	0.222 ± 0.151	P = 0.166
Fear of COVID-19 score < 50	0.187 ± 0.174	U = 1,010

Bold values mean statistical significant differences.

### 3.4 Passively generated data and COVID-19 vaccination status

Next, we evaluated the association between COVID-19 vaccination status and passively generated data streams (Table 4). Patients who were fully vaccinated and partially vaccinated, on average, visited more places of interest compared to patients who were not vaccinated (*p* = 0.018).

**Table 4:** Statistical significance difference in activity characteristics based on COVID-19 vaccination.

Variables of physical and social activity	Mean ± SD	Statistical value
Number of POI		
Fully vaccinated	2.908 ± 0.985	**P = 0.018**
Partially vaccinated	3.837 ± 0.983	KW = 7.998
Unvaccinated	2.704 ± 0.7353	
Distance to home (km)		
Fully vaccinated	43.498 ± 171.353	P = 0.848
Partially vaccinated	7.588 ± 13.461	KW = 0.330
Unvaccinated	7.829 ± 9.727	
Time spent at home ratio (range [0;1])		
Fully vaccinated	0.688 ± 0.255	P = 0.548
Partially vaccinated	0.654 ± 0.201	KW = 1.2
Unvaccinated	0.723 ± 0.088	
Activity duration ratio (range [0;1])		
Fully vaccinated	0.561 ± 0.144	P = 0.671
Partially vaccinated	0.548 ± 0.153	KW = 0.798
Unvaccinated	0.608 ± 0.108	
Movement duration ratio (range [0;1])		
Fully vaccinated	0.101 ± 0.049	P = 0.148
Partially vaccinated	0.092 ± 0.042	KW = 3.821
Unvaccinated	0.062 ± 0.041	
Screen time ratio (range [0;1])		
Fully vaccinated	0.159 ± 0.113	P = 0.911
Partially vaccinated	0.156 ± 0.098	KW = 0.186
Unvaccinated	0.151 ± 0.058	

Bold values mean statistical significant differences.

### 3.5 Trends of activity and sociability

**Figure 2:**
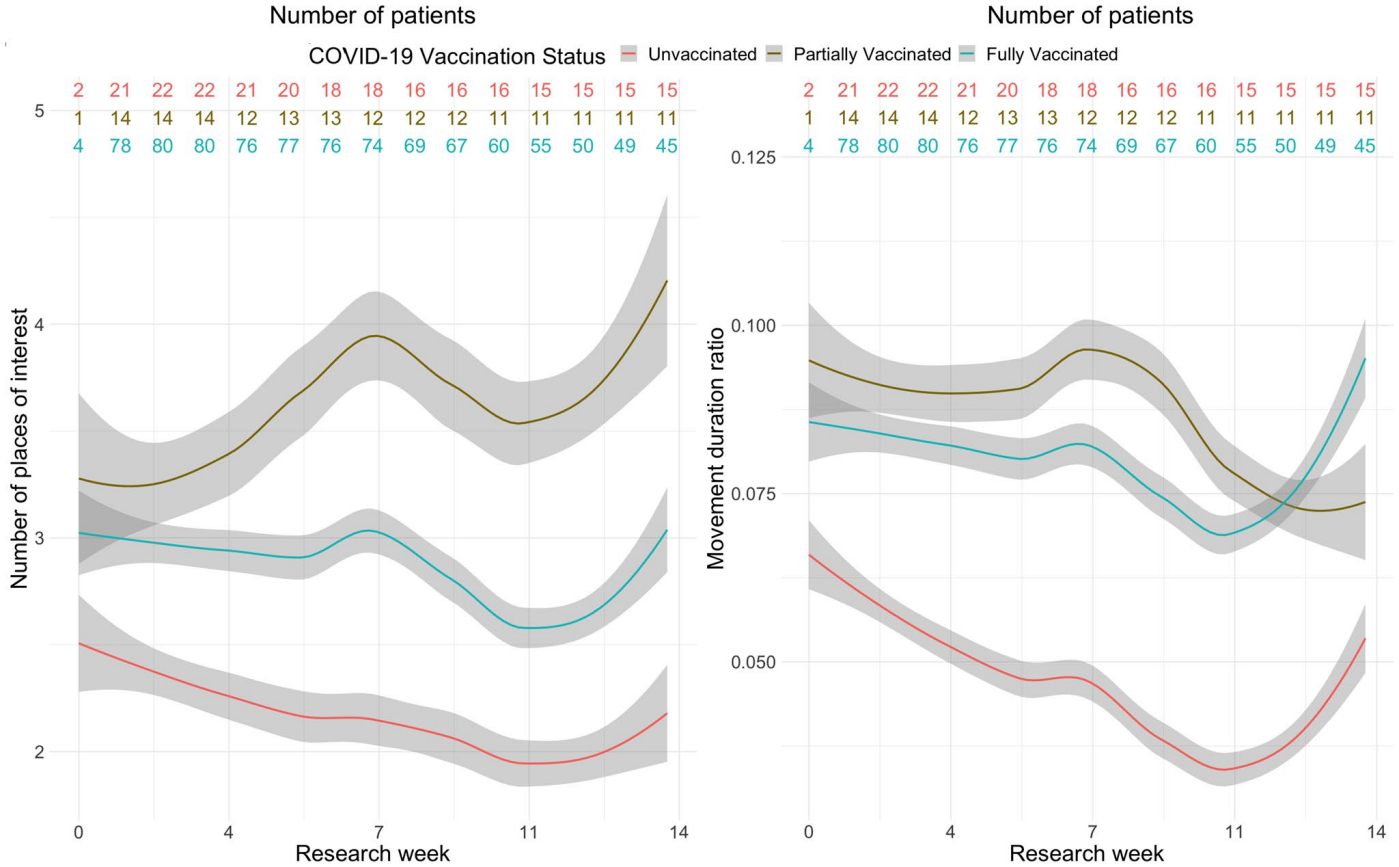
A plot of daily number of POI and moving duration by COVID 19 vaccination status.

The local polynomial regression model, which is a generalization of the moving average and polynomial regression, was applied to analyze whether passively collected data on activity and sociability had different trends between patients who fear COVID-19 and those who do not, as well as patients with different COVID-19 vaccination status. The fitted values were complemented by a 95% confidence interval. Since this model is non-parametric, the results are best interpreted visually. It was observed that the groups of patients with and without fear of COVID-19 did not differ in activity and sociability. Significant differences were observed in patients with different vaccination status. The models with significant differences between these groups are shown in Figure 2 (the shaded regions indicate 95% confidence intervals). It can be noticed that, although at the beginning of the study, all patients had a similar number of POI, after a week, this number in partially vaccinated patients increased and remained on average around four places. Meanwhile, fully vaccinated or unvaccinated patients had a similar trend of POI, and it did not exceed three visits per day during the entire treatment period. Unvaccinated patients reached the maximum number of POI at the beginning of the study (2.5 POI, CI [2.3; 2.6]), while the minimum number of POI, of 2 (CI [1.8; 2.1]), was reported in the 11th week. Partially vaccinated patients had the maximum number of POI in the 14th week of the trial (4 POI, CI [3.8; 4.5]), and the minimum number of 3.3 POI (CI [2.9; 3.6]) was registered at the beginning of the trial. Vaccinated patients had the maximum number of POI in the 14th week of the trial (3 POI, CI [2.8; 3.2]), and the minimum number of POI in the 11th week of the trial (2.6 POI, CI [2.5; 2.7]). The ratio of movement duration significantly differs between unvaccinated patients and partially or fully vaccinated patients. Unvaccinated patients had the maximum movement duration ratio (0.065, Cl [0.06; 0.07]) at the beginning of the trial, while the minimum was in the 10th week of the trial (0.03; Cl [0.025; 0.035]). Partially vaccinated patients reached the maximum movement duration ratio in the 7th week of the trial (0.095, Cl [0.09; 0.1]) and the minimum in the 14th week of the trial (0.075, Cl [0.07; 0.08]). Vaccinated patients had the maximum movement duration ratio in the 14th week of the trial (0.095, Cl [0.085; 0.1]) and the minimum in the 10th week of the trial (0.065, Cl [0.06; 0.07]).

## 4 Discussion

In this prospective study, we found that fear of COVID-19 and vaccination status were associated with the behavior of cancer patients in their natural home environment, as measured using sensors of personal smartphones. A higher perceived fear of COVID-19 was associated with less movement, more time spent at home, less time spent outside of home, and a lower number of visited places. Unvaccinated patients visited fewer places and were moving less overall during a 14-week follow-up when compared to vaccinated patients.

We found that greater perceived fear of COVID-19 was associated with longer time spent at home, less time spent outside of home, and a lower number of visited places, suggesting that perceived fear of COVID-19 infection continues to have an adverse effect on the behavior of cancer patients even after the COVID-19 pandemic. Studies performed during the COVID-19 pandemic demonstrated that patients with cancer are vulnerable to experiencing fear related to the COVID-19 pandemic, which was associated with adverse mental health outcomes (18, 19). Quarantine, stay-at-home orders, wider adoption of telehealth, and other public health measures were important during the pandemic to prevent disease spread and protect vulnerable patients with cancer. Post-pandemic and re-entry anxiety can also contribute to greater social isolation of patients with cancer in the immediate post-pandemic period. Social isolation and loneliness are associated with a worse quality of life and prognosis in cancer patients (28). Our findings suggest that perceived fear of COVID-19 can be an important barrier, limiting the mobility and social interactions of patients with cancer. Further studies are needed to better understand the lingering fear and adverse psychological and behavioral consequences of the COVID-19 pandemic on cancer patients to improve their social functioning and quality of life.

Unvaccinated patients visited fewer places and were moving less overall during the 14-week follow-up as compared to vaccinated patients. Immunocompromised and unvaccinated status can limit social behaviors due to safety concerns related to severe SARS-CoV-2 infection, and it has been shown that vaccination for COVID-19 is associated with improved social interaction after vaccination. For example, a study with 220 patients with thoracic cancer using self-report questionnaires found that, after vaccination for COVID-19, patients increased contact with family and friends, use of public transport, and grocery shopping (29). Another study with 274 immunocompromised patients, with the most common diagnoses of rheumatic diseases, inflammatory bowel diseases, and psoriasis, found that social participation, which was measured using the Index for the Assessment of Health Impairments, increased after COVID-19 vaccination (30). Surveys in the general population found improved psychological well-being and changes in preventive behaviors after COVID-19 vaccination (31, 32). To the best of our knowledge, this was the first study to demonstrate the impact of COVID-19 status on the behavior of patients with cancer in their home environments using personal smartphone data, suggesting that this approach can be used to monitor the social behavior of patients with cancer.

Most patients in our cohort were fully or partially vaccinated and only 16% of patients were not vaccinated. COVID-19 vaccine uptake in our study was comparable to a previous study that used American Society of Clinical Oncology Registry data (33), revealing that younger age, diagnosis of a metastatic tumor or non-B-cell hematologic malignancies, and co-morbidities were associated with lower vaccine uptake. In our study, non-vaccinated status was higher in patients with prior COVID-19 infection and cervical cancer. COVID-19 vaccines are safe in patients with cancer; however, their effectiveness can be lower in patients with certain cancers, such as hematological malignancies, and in those undergoing certain treatments (34–36), such as B cell depletion. It is important to continue educating patients with cancer about the safety and effectiveness of COVID-19 vaccination given ongoing breakthrough infections and a prediction that COVID-19 will become endemic in the near future.

Limitations of this study include heterogeneous sample size with regard to cancer types and treatment, given that cancer symptoms, location, and treatment can have an impact on the mobility of cancer patients. While we included patients with good functional status, it is possible that some patients experienced disease progression and/or treatment complications. Data used for the study were generated with various smartphone sensors because we used personal smartphone devices, which can impact the quality of the data. We also did not formally test the digital and health literacy of patients before enrollment in the study; however, we used passively generated data that did not require patient input and we expected adequate digital literacy from an individual owning a personal smartphone.

## 5 Conclusion

The COVID-19 pandemic continues to have an impact on the behavior of patients with cancer even after the termination of the global pandemic. Fear of COVID-19 infection and vaccination status are associated with the behavior of patients with cancer, as measured using passively generated smartphone sensor data in their natural home environment. Higher perceived fear of COVID-19 was associated with less movement, more time spent at home, and less time spent outside of home as well as a lower number of visited places. Unvaccinated patients visited fewer places and were moving less overall during a 14-week follow-up compared to vaccinated patients.

## Data availability statement

The raw data supporting the conclusions of this article will be made available by the authors, without undue reservation.

### Code availability

Data were analyzed using Python (version 3.10.9) and R (version 4.2.2). The code used for the analysis is available upon reasonable request from the corresponding author for non-commercial purposes.

## Ethics statement

The studies involving humans were approved by the KAUNAS REGIONAL BIOMEDICAL RESEARCH ETHICS COMMITTEE. The studies were conducted in accordance with the local legislation and institutional requirements. The participants provided their written informed consent to participate in this study.

## Author contributions

GK: Data curation, Formal analysis, Writing – original draft, Writing – review & editing. GJ: Data curation, Formal analysis, Writing – original draft. NŠ: Data curation, Formal analysis, Writing – original draft. IB: Writing – review & editing, Project administration, Writing – original draft. EK: Investigation, Validation, Writing – original draft, Writing – review & editing, Data curation. DV: Investigation, Validation, Writing – original draft, Writing – review & editing. AI: Investigation, Validation, Writing – original draft, Writing – review & editing. LJ: Investigation, Validation, Writing – original draft, Writing – review & editing. RB: Software, Writing – original draft. RK: Formal Analysis, Validation, Writing – original draft, Writing – review & editing. TK: Conceptualization, Funding acquisition, Validation, Writing – original draft, Writing – review & editing. EJ: Supervision, Validation, Writing – original draft, Writing – review & editing. AB: Conceptualization, Investigation, Methodology, Supervision, Validation, Writing – original draft, Writing – review & editing.

## Footnotes

^1^https://laima2.proit.lt

^2^https://www.Beiwe.org/
